# Role of Bevacizumab on Vascular Endothelial Growth Factor in Apolipoprotein E Deficient Mice after Traumatic Brain Injury

**DOI:** 10.3390/ijms23084162

**Published:** 2022-04-09

**Authors:** Tiziana Genovese, Daniela Impellizzeri, Ramona D’Amico, Roberta Fusco, Alessio Filippo Peritore, Davide Di Paola, Livia Interdonato, Enrico Gugliandolo, Rosalia Crupi, Rosanna Di Paola, Salvatore Cuzzocrea, Marika Cordaro, Rosalba Siracusa

**Affiliations:** 1 Department of Chemical, Biological, Pharmaceutical and Environmental Sciences, University of Messina, 98166 Messina, Italy; tiziana.genovese@unime.it (T.G.); dimpellizzeri@unime.it (D.I.); rdamico@unime.it (R.D.); alessiofilippo.peritore@unime.it (A.F.P.); dipaolad@unime.it (D.D.P.); livia.interdonato@unime.it (L.I.); rsiracusa@unime.it (R.S.); 2 Department of Clinical and Experimental Medicine, University of Messina, 98125 Messina, Italy; rfusco@unime.it; 3 Department of Veterinary Science, University of Messina, 98168 Messina, Italy; egugliandolo@unime.it (E.G.); rcrupi@unime.it (R.C.); 4 Department of Pharmacological and Physiological Science, School of Medicine, Saint Louis University, Saint Louis, MO 63104, USA; 5 Department of Biomedical, Dental and Morphological and Functional Imaging, University of Messina, 98125 Messina, Italy; marika.cordaro@unime.it

**Keywords:** traumatic brain injury, blood–brain barrier, neuroinflammation, vascular endothelial growth factor, vascular risk

## Abstract

Traumatic brain injury (TBI) disrupts the blood–brain barrier (BBB). Vascular endothelial growth factor (VEGF) is believed to play a key role in TBI and to be overexpressed in the absence of apolipoprotein E (ApoE). Bevacizumab, a VEGF inhibitor, demonstrated neuroprotective activity in several models of TBI. However, the effects of bevacizumab on Apo-E deficient mice are not well studied. The present study aimed to evaluate VEGF expression and the effects of bevacizumab on BBB and neuroinflammation in ApoE^−/−^ mice undergoing TBI. Furthermore, for the first time, this study evaluates the effects of bevacizumab on the long-term consequences of TBI, such as atherosclerosis. The results showed that motor deficits induced by controlled cortical impact (CCI) were accompanied by increased brain edema and VEGF expression. Treatment with bevacizumab significantly improved motor deficits and significantly decreased VEGF levels, as well as brain edema compared to the control group. Furthermore, the results showed that bevacizumab preserves the integrity of the BBB and reduces the neuroinflammation induced by TBI. Regarding the effects of bevacizumab on atherosclerosis, it was observed for the first time that its ability to modulate VEGF in the acute phase of head injury prevents the acceleration of atherosclerosis. Therefore, the present study demonstrates not only the neuroprotective activity of bevacizumab but also its action on the vascular consequences related to TBI.

## 1. Introduction

The global incidence of traumatic brain injury (TBI) is estimated at 939 cases per 100,000 people; therefore, approximately 69.0 million people worldwide will suffer from TBI each year [1].

TBI, in most cases, occurs as a result of falls, motor vehicle or sports accidents and consists of immediate damage to the brain. This develops in two stages called primary damage and secondary damage [2]. The first is induced by a direct insult to the head that leads to brain contusion and hemorrhage in the area of the insult. Later, the brain damage spreads from the bruised area to the surrounding areas. During secondary damage, the breakdown of the blood–brain barrier (BBB) typically occurs, which, under physiological conditions, has the function of limiting the inflow of intravascular contents [3]. In pathological conditions, such as following a TBI, the interruption of the BBB leads to cerebral edema through an excessive accumulation of fluid in the brain and the infiltration of pro-inflammatory cells in the brain parenchyma [4,5]. Therefore, the protection and/or recovery of BBB function could be a promising therapeutic target for the resolution of TBI-induced secondary damage [6,7]. Numerous studies highlight the excessive production of factors responsible for the increase in vascular permeability in damaged nerve tissues, such as reactive oxygen species (ROS), matrix metalloproteinases (MMPs), angiogenic factors, inflammatory cytokines, leukocyte adhesion and immune cell extravasation.

This involves the decrease of endothelial cells due to apoptosis and of the tight junctions (TJ) with consequent alteration of the function of the BBB [8,9,10]. The role of vascular endothelial growth factor (VEGF) and matrix metalloproteinase (MMP) is highlighted in animal models of TBI, the inhibition of which appears to improve the disruption of BBB function [11,12,13]. Bevacizumab is a known inhibitor of VEGF-A, a VEGF isoform that stimulates the proliferation and migration of endothelial cells. The specific binding of bevacizumab to the VEGF-A protein reduces the levels of the VEGF protein by limiting the formation of new blood vessels. Furthermore, it also has an anti-inflammatory function as it reduces the activity of macrophages and inhibits the infiltration of inflammatory elements in the brain that cause neuronal damage. The anti-angiogenic role of bevacizumab in tumors is well studied [14]. It is used in the treatment of gliomas [15] and colon cancer [16], and recently, a beneficial effect was also observed in macular edema in the sensory retina of diabetic patients [17].

TBI is often associated with atherosclerosis and cardiovascular mortality in humans [18,19,20]. As is well known, TBI is the result of mechanical forces applied to the skull, which are transmitted to the brain. This can lead to widespread brain damage and systemic complications. In particular, oxidative stress and inflammatory processes related to TBI could become chronic and lead to secondary problems such as cardiovascular ones [21]. Therefore, the aim of this study was to first evaluate whether bevacizumab administered in the first phase after TBI was able to act on BBB damage and inflammatory processes by blocking VEGF. We also chose to carry out the TBI experimental model on apolipoprotein E-deficient (ApoE^−/−^) mice with atherosclerosis to observe the long-term effects of TBI on this pathology.

## 2. Results

### 2.1. Effect of Bevacizumab on Behavioral Function after TBI

**Figure 1:**
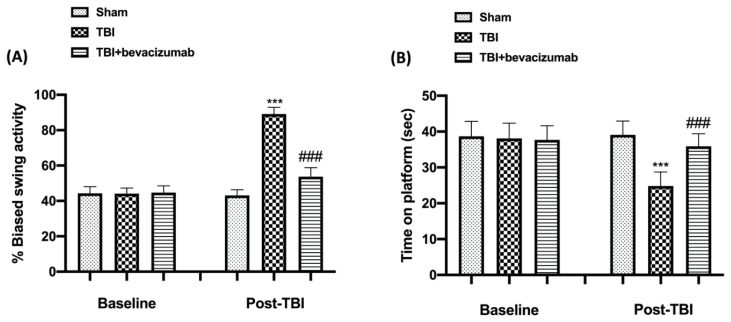
Bevacizumab helps recovery and improves behavioral function. At 24 h after TBI, mice showed significant impairments in motor deficits as revealed by appreciably biased swing activity (**A**) and reduced time to stay on rotarod (**B**). On the contrary, treatment with bevacizumab 1 h post-TBI significantly improved motor function evaluated by EBST (**A**) and Rotarod test (**B**). All data are expressed as mean ± SD from n = 5 mice for each group. *** *p* < 0.001 vs. Sham; ### *p* < 0.001 vs. TBI.

Behavioral tests were performed to assess the relationship between neurological deficits and TBI. In particular, we performed the EBST and the Rotarod test to evaluate motor functions. Mice that suffered head trauma showed alterations in locomotor functions as shown in Figure 1A,B. Bevacizumab treatment improved latency compared to the TBI group.

### 2.2. Bevacizumab Reduces Edema and the Severity of Brain Trauma

**Figure 2:**
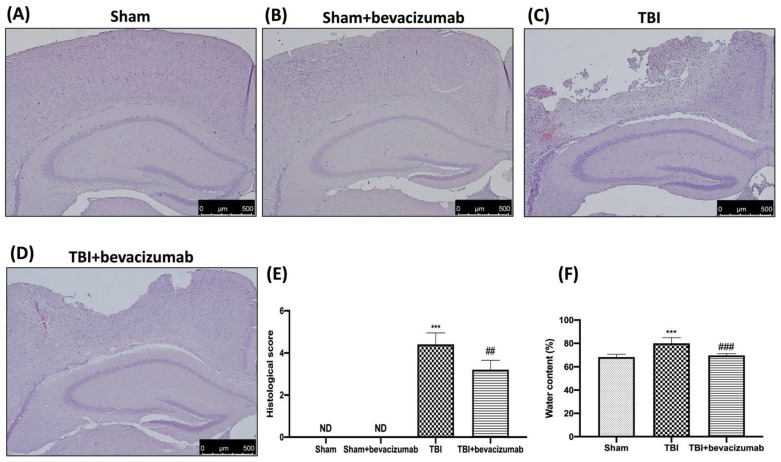
Bevacizumab reduces both edema and histological score. A histological investigation of brain sections showed a intact tissue structure in Sham (**A**) and Sham + bevacizumab (**B**) mice respect tissue disorganization and cell infiltration present in the TBI group (**C**). Significant protection from the TBI was apparent in bevacizumab-treated mice (**D**). The histological score was measured (**E**). Brain edema was measured by brain water content in the ipsilateral hemisphere. At 24 h after, TBI showed an increase in levels of water content in the TBI brain (**F**), while the treatment with bevacizumab decreased the water content in the TBI brain (**F**). Images are figurative of at least 3 independent experiments. Values = means ± SD of 5 animals per group; *** *p* < 0.001 vs. Sham; ## *p* < 0.01 vs. TBI; ### *p* < 0.001 vs. TBI.

To study the severity of the trauma in the perilesional area, histopathological analysis was carried out using H&E staining. The TBI group showed significant tissue damage and numerous eosinophilic neurons (Figure 2C,E) compared to the brain of the Sham and Sham + bevacizumab groups (Figure 2A,B,E), both in the perilesional area and in the white matter. Bevacizumab treatment significantly reduced the degree of brain injury compared with the vehicle group (Figure 2D,E).

The water content in the brain is a sensitive measure of brain edema. This finding indicates a pathology associated with endothelial cell activation and endothelial dysfunction. As shown in Figure 2, the water content was significantly increased in animals undergoing TBI compared with the Sham group. This increase was significantly reduced by bevacizumab treatment 24 h after injury (Figure 2F).

### 2.3. Effect of Bevacizumab on VEGF and BBB Integrity

**Figure 3:**
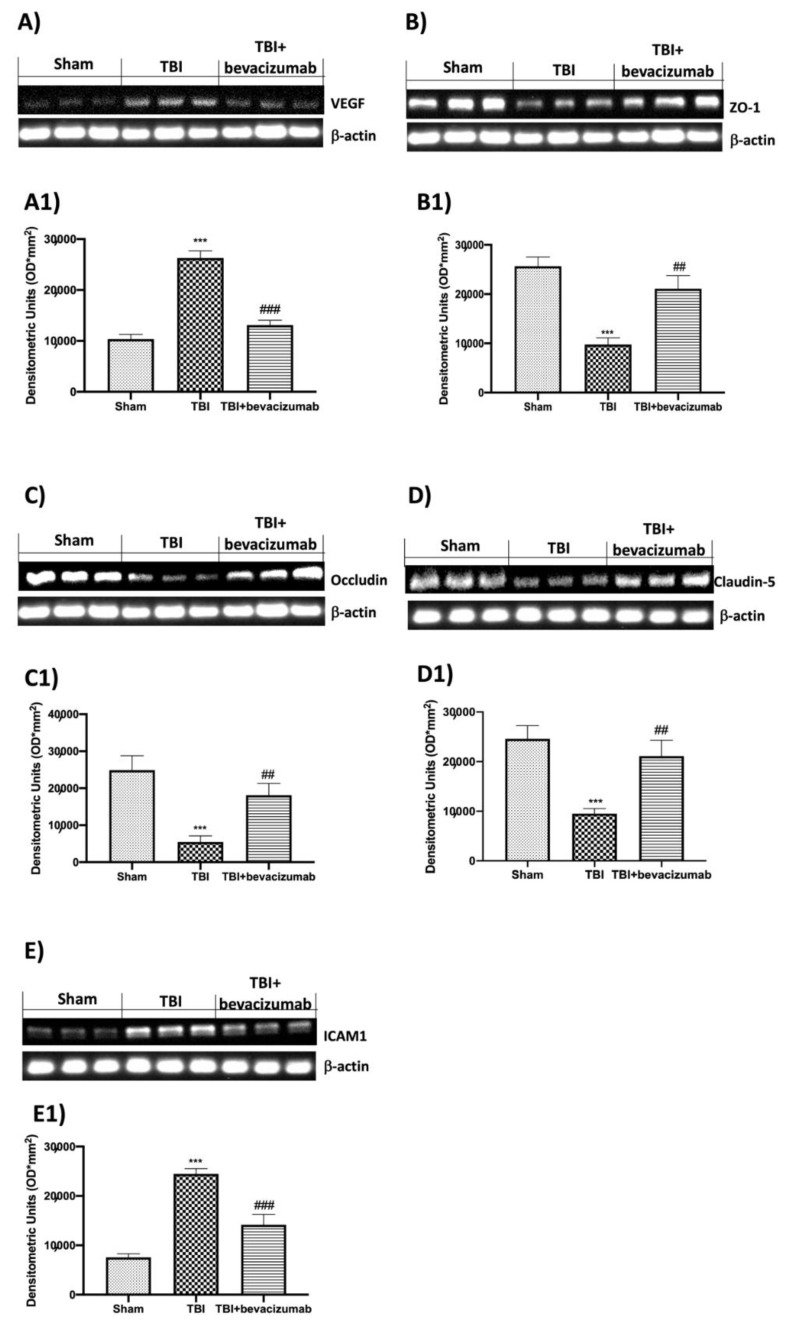
Effects of bevacizumab on VEGF, TJs and ICAM1 expression 24 h after TBI. TBI activates the VEGF (**A**,**A1**) protein and reduces the expression of ZO-1 (**B**,**B1**), Occludin (**C**,**C1**), Claudin-5 (**D**,**D1**) and ICAM1 (**E**,**E1**). On the other hand, treatment with bevacizumab limits the expression of VEGF (**A**,**A1**) and the alteration of ZO-1 (**B**,**B1**), Occludin (**C**,**C1**), Claudin-5 (**D**,**D1**) and ICAM1 (**E**,**E1**). Data show one representative blot from three independent experiments with similar results. Data are expressed as mean ± SD from n = 5 mice/group. *** *p* < 0.001 vs. Sham; ## *p* < 0.01 and ### *p* < 0.001 vs. TBI.

To evaluate the ability of bevacizumab to modulate VEGF expression, thereby protecting BBB, we performed Western blot analysis to evaluate the expression of TJs. The investigation showed a significant increase in VEGF 24 h after TBI induction, compared with the control group, while bevacizumab treatment significantly reduced VEGF expression (Figure 3A,A1). To determine the BBB alterations, we evaluated the expression of ZO-1, Occludin and Claudin-5 by Western blot analysis (Figure 3B,B1,C,C1,D,D1, respectively). The examined TJs were strongly reduced in the TBI group compared to the Sham group. Administration of bevacizumab limited the BBB alteration by reducing the loss of TJs. Furthermore, our analysis revealed the action of bevacizumab on ICAM1 expression that was significantly reduced compared to the TBI group (Figure 3E,E1).

### 2.4. Bevacizumab Modulates NF-kB Pathway after TBI

**Figure 4:**
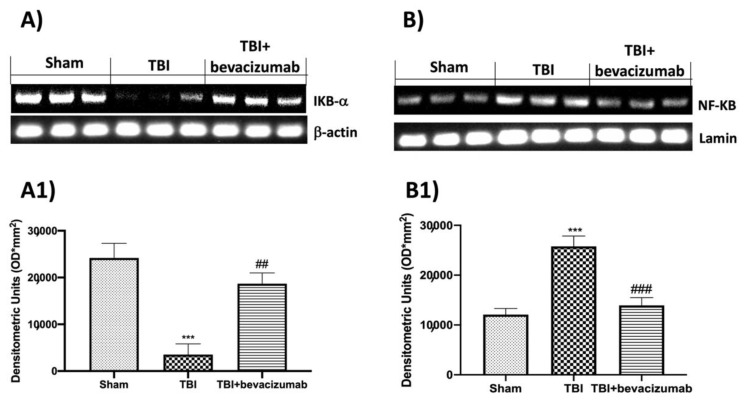
Effects of bevacizumab on NF-kB pathway 24 h after TBI. Degradation of IKB-α was significantly increased after TBI (**A**,**A1**). Additionally, TBI resulted in enhanced NF-kB (**B**,**B1**), whereas bevacizumab treatment significantly restored IkB-α levels (**A**,**A1**) and reduced NF-kB expression (**B**,**B1**). Data show one representative blot from three independent experiments with similar results. Data are expressed as mean ± SD from n = 5 mice/group. *** *p* < 0.001 vs. Sham; ## *p* < 0.01 and ### *p* < 0.001 vs. TBI.

To understand the effect of bevacizumab on neuroinflammation in the progression of TBI, we evaluated the NF-kB pathway through Western blot analysis. Our results revealed a significant decrease in IkB-α expression in TBI-damaged mice compared with the Sham group. Bevacizumab treatment restored IkB-α levels (Figure 4A,A1). The reduced expression of IkB-α in the nuclear fraction of the control group and the contrary increase in the TBI-injured group confirmed the bevacizumab prevention of IkB-α cytosolic degradation. Moreover, bevacizumab treatment noticeably decreased the levels of NF-kB compared to the increase induced by TBI; instead, to verify the nuclear translocation of NF-kB, we performed an evaluation of NF-kB in the cytosolic fraction of the samples (Figure 4B,B1).

### 2.5. Bevacizumab Reduced Inflammatory Response and Astrocytes and Microglia Activation

**Figure 5:**
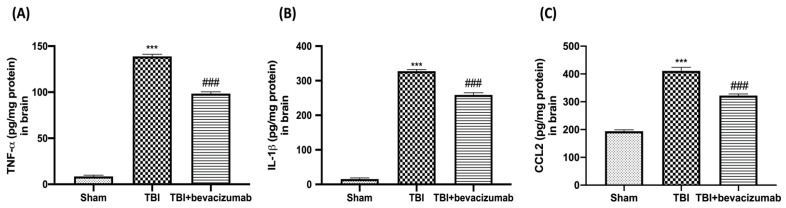
Effects of bevacizumab on TNF-α, IL-1β and CCL2 in the brain after TBI. TNF-a, IL-1b and CCL2 levels were quantified by ELISA in the brain tissue lysate 24 h after TBI. Results show significantly increased levels of TNF-a (**A**), IL-1b (**B**) and CCL2 (**C**) in the brains of TBI mice compared with Sham mice. Bevacizumab treatment significantly restored the levels of these pro-inflammatory cytokines and chemokines. Data are expressed as mean ± SD from n = 5 mice/group. *** *p* < 0.001 vs. Sham; ### *p* < 0.001 vs. TBI.

To evaluate the anti-inflammatory and neuroprotective effect of bevacizumab treatment, we performed ELISA analysis for TNF-α, IL-1β and CCL2. We demonstrated that TNF-α, IL-1β and CCL2 levels were significantly increased after TBI (Figure 5A–C, respectively) compared with the Sham group (Figure 5A–C, respectively). Bevacizumab treatment significantly reduced TNF-α (Figure 5A), IL-1β (Figure 5B) and CCL2 (Figure 5C) production.

**Figure 6:**
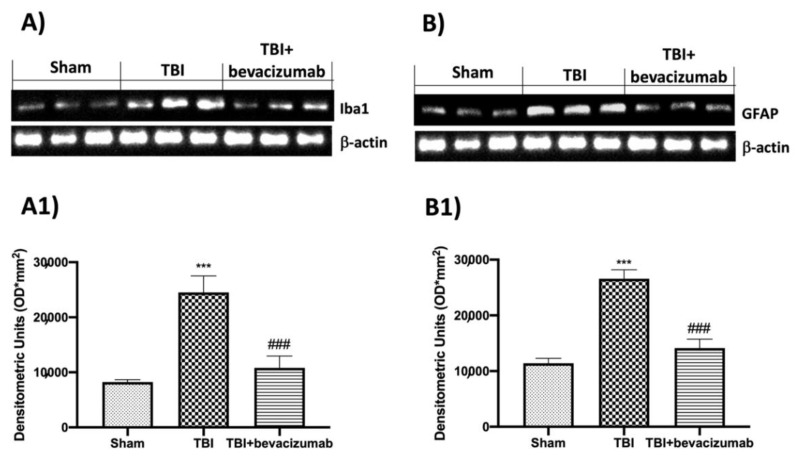
Effects of bevacizumab on Iba1 and GFAP 24 h after TBI. A remarkable increment in microglia and astrocytes activation was observed by Western blot analysis for Iba1 (**A**,**A1**) and GFAP (**B**,**B1**), respectively, in TBI-injured animals, compared to the Sham group (**A**,**A1**,**B**,**B1**). Treatment with bevacizumab reduced the activation of both microglia (**A**,**A1**) and astrocytes (**B**,**B1**). Data show one representative blot from three independent experiments with similar results. Data are expressed as mean ± SD from n = 5 mice/group. *** *p* < 0.001 vs. Sham; ### *p* < 0.001 vs. TBI.

Astrocytes and microglia activation are key components of the progression of TBI. Thus, we evaluated, by Western blot analysis, the expression of Iba1 and GFAP as a marker of microglial and astrocyte activation, respectively. A considerable increase in Iba1 and GFAP expressions (Figure 6A,B respectively; see graphs A1 and B1) was observed in mice of the TBI group compared with the Sham group (Figure 6 A,B, respectively; see graphs A1 and B1), whereas astrogliosis and microgliosis were appreciably decreased by bevacizumab (Figure 6A,B, respectively; see Figure 6A1,B1).

### 2.6. Effects of Bevacizumab Treatment on Apoptosis Pathway after TBI

**Figure 7:**
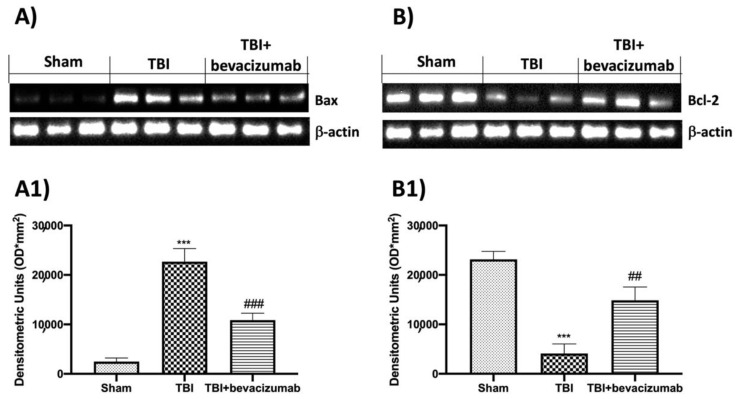
Effects of bevacizumab on Bax and Bcl-2 24 h after TBI. Representative Western blots for Bax (**A**,**A1**) and Bcl-2 (**B**,**B1**) expression were performed. Data show one representative blot from three independent experiments with similar results. Data are expressed as mean ± SD from n = 5 mice/group. *** *p* < 0.001 vs. Sham; ## *p* < 0.01 and ### *p* < 0.001 vs. TBI.

The role of Bax and Bcl-2, a pro- and anti-apoptotic factor, respectively, was considered by Western blot analysis. The expression of Bax was markedly increased in the TBI group compared to the Sham mice. On the contrary, bevacizumab treatment prevented TBI-induced Bax expression (Figure 7A,A1). The levels of Bcl-2 were rapidly reduced after TBI, and treatments with bevacizumab showed an increase in Bcl-2 expression (Figure 7B,B1).

### 2.7. Effects of Bevacizumab on Atherosclerosis

**Figure 8:**
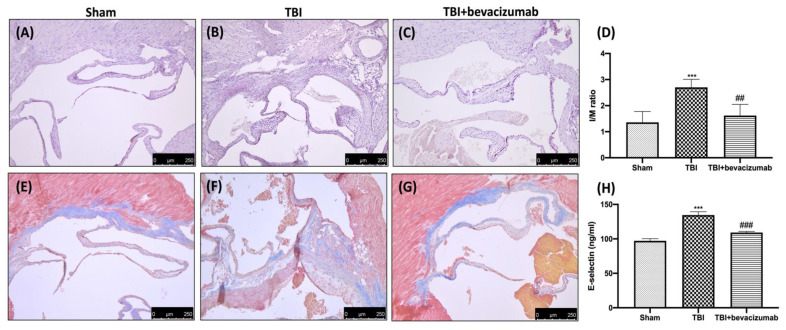
Effects of bevacizumab on E-selectin and atherosclerosis. Representative images of Sham (**A**), TBI (**B**) and TBI+bevacizumab (**C**) and quantification of lesion area normalized by the respective medial area of the aorta at aortic root stained with H&E (**D**) 6 weeks following TBI. Fibrosis evaluation (Masson’s trichrome stain): Sham (**E**), TBI (**F**), and TBI+bevacizumab (**G**). Plasma soluble E-selectin levels were measured 6 weeks following TBI by ELISA kit. Results show a significantly increased level of E-selectin in plasma of TBI mice compared with Sham mice. Bevacizumab treatment significantly restored the levels of E-selectin (**H**). Data are expressed as mean ± SD from n = 5 mice/group. *** *p* < 0.001 vs. Sham; ## *p* < 0.01 and ### *p* < 0.001 vs. TBI.

Cross-sectional analysis of the atherosclerotic plaque area at the aortic valve level showed an increase in plaque area in the TBI group (Figure 8B,D) compared to the Sham group (Figure 8A,D), while treatment with bevacizumab prevented the worsening of atherosclerosis (Figure 8C,D). Masson’s trichrome stain revealed a noticeable increase in fibrosis in the hearts of the TBI group (Figure 8F) compared to the Sham group (Figure 8E). The degree of fibrosis (blue-colored fibrotic area) was reduced by bevacizumab treatment (Figure 8G). To determine whether systemic pro-inflammatory changes occur following TBI and whether these are associated with increased vascular disease, we assessed the plasma levels of E-selectin, and subsequently, atherosclerosis was assessed by histological analysis. The results obtained by the ELISA kit showed an increase in plasma E-selectin levels 6 weeks after TBI. This indicates an increase in circulating neutrophils which, in turn, induce greater endothelial adhesiveness. Bevacizumab treatment was able to contain the increase in E-selectin levels (Figure 8H).

## 3. Discussion

The damaging effects of TBI often extend beyond the damage zone. Moderate to severe TBI induces several inflammatory signals, which increase the release of pro-inflammatory cytokines and chemokines, resulting in monocyte activation and infiltration, glial activation, neuronal loss and persistent inflammation [22,23]. Inflammation can become chronic and contribute to increased medical morbidity. TBI is associated with atherosclerosis and cardiovascular mortality in humans [24]. In particular, it was observed that patients with TBI, both men and women, have a significantly higher risk of coronary artery disease than those without TBI [25,26]. A veteran clinical study has shown that TBI can promote a state of chronic inflammation that could promote vascular complications and accelerate atherosclerosis [27]. Therefore, the inflammatory mechanisms between TBI and coronary heart disease appear to be linked, but actual evidence for this association is still limited. ApoE-deficient mice are widely used to study the factors involved in atherosclerosis. In a preclinical study conducted on these mice, it was observed that TBI causes an acceleration of atherosclerosis [27]. This phenomenon is linked to an increase in the number of monocytes in peripheral blood 6 weeks after TBI compared to the control group. Therefore, based on this information present in the literature, our hypothesis was that bevacizumab, thanks to its anti-inflammatory activity, was able to act on the inflammatory response induced by TBI within 24 h and thus prevent the persistence and propagation of inflammation beyond the damage zone. In particular, for the first time, we wanted to demonstrate that the use of bevacizumab in the acute phase of TBI could prevent the acceleration of atherosclerosis, as Jintao Wang et al. [27] observed in their study on ApoE-deficient mice. Bevacizumab is a known VEGF inhibitor. This factor is known to play a crucial role in TBI. Elevated levels of VEGF in serum and cerebrospinal fluid are found in patients with TBI [28,29,30,31]. The overexpression of VEGF can compromise the BBB and consequently cause a greater infiltration of cells and inflammatory factors such as cytokines and chemokines, which aggravate cerebral edema and increase the risk of secondary consequences such as cognitive and motor deficits [30].

Consistent with literature data, our results demonstrated VEGF overexpression 24 h after TBI. This event led to the interruption of the BBB demonstrated by the reduction of TJs such as ZO-1, Occludin and Claudin-5. Treatment with bevacizumab, on the other hand, inhibited the upregulation of VEGF and consequently prevented the reduction of TJs.

VEGF upregulation was shown to affect not only TJs but also ICAM-1 expression in the central nervous system, brain endothelial cell cultures and retinal endothelium [32,33]. Our results showed an increase in ICAM-1 24 h after TBI, while bevacizumab treatment reduced ICAM-1 expression in the brains of ApoE-deficient mice 24 h after TBI. The increased permeability of BBB results in the extensive infiltration of immune cells and the promotion of neuroinflammation [34,35]. In this regard, we wanted to evaluate the NF-kB pathway, the activation of immune cells such as astrocytes and microglia and the levels of pro-inflammatory cytokines and chemokines released following TBI. Our results demonstrated that TBI induces a significant reduction of IKB-a at the cytoplasmic level and a consequent increase of NF-kB at the nuclear level. Furthermore, 24 h after TBI, we observed a notable increase in specific markers for astrocytes and microglia such as GFAP and Iba-1. To conclude, we also found an increase in the release of cytokines and chemokines such as IL-1b, TNF-a and CCL2. The results obtained after treatment with bevacizumab demonstrated the ability of this drug to primarily reduce the degradation of IKB-a and the nuclear translocation of NF-kB 24 h after TBI. In addition, the treatment also significantly reduced the expression of GFAP, Iba-1 and the release of pro-inflammatory cytokines and chemokines. The apoptotic process is a type of programmed cell death activated following head trauma [36,37]. Therefore, limiting this process is a critical step in treating head trauma. In the present study, we evaluated whether treatment with bevacizumab was able to mitigate the apoptotic process induced by head trauma, evaluating the expression of two factors involved in the regulation of the apoptotic process such as Bax, responsible for the development of neuronal death [38], and Bcl-2 protective against neuronal cells [39,40]. Our results showed that after TBI, there is an increase in Bax expression and a significant decrease in Bcl-2, which highlights an imbalance in the apoptotic pathway. Bevacizumab treatment was able to significantly reduce Bax levels by returning Bcl-2 expression to levels in the Sham group. At this point, after demonstrating the ability of bevacizumab to reduce the breakdown of the BBB by blocking the overexpression of VEGF and modulating the neuroinflammation induced by TBI, we moved on to the evaluation of the long-term effects of TBI and our treatment on atherosclerosis. Ours are, for the moment, preliminary data that will have to be followed by other studies for the evaluation of the mechanisms underlying the association between TBI and atherosclerosis. Selectins are a group of adhesion receptors that play a role in leukocyte capture, rolling and firm adhesion, especially under the increased shear stress of arteries [41]. The deletion of P-selectin and E-selectin decreases atherosclerosis in mouse models [42]. In addition, E-selectin is expressed in the endothelium of human atherosclerotic lesions and in plasma [43,44]. Therefore, E-selectin is believed to be an important player in the formation of lesions. Our results obtained at 6 weeks from TBI showed that mice with TBI have elevated levels of soluble E-selectin in plasma, which represents a specific endothelial biomarker for adhesive interactions of the endothelium of leukocytes [45]. This evidence provides evidence for the causal relationship between head injury and subsequent vascular complications. Furthermore, another important fact highlighted by our histopathological analysis was the demonstration that bevacizumab can prevent an acceleration of atherosclerosis. In particular, our results showed an increase in plaque area in mice with TBI compared to the Sham group after 6 weeks from TBI, while treatment with bevacizumab preserved the increase in the size of the aortic valve.

In summary, our study showed, for the first time, that the treatment of TBI with bevacizumab is able first of all to reduce the inflammatory processes induced by TBI after 24 h and subsequently prevent vascular consequences probably related to the chronicization of inflammation.

Therefore, we can conclude that targeting inflammatory pathways in TBI patients can reduce subsequent vascular complications.

## 4. Materials and Methods

### 4.1. Animals

Male ApoE^−/−^ mice on the C57BL6/J strain background (Jackson Laboratory, Bar Harbor, Maine), aged 8 weeks, were purchased for the study. Mice were housed in a controlled location (under specific pathogen-free conditions, at 22 ± 1 °C and with a 12:12 h light/dark cycle) with tap water ad libitum. At 10 weeks of age, mice were started on a Western diet (TD88137, Harlan, WI, USA) and at 14 weeks of age, mice were arbitrarily distributed to the TBI or Sham procedures.

The study was permitted by the University of Messina Review Board for the care of animals. All animal experiments were performed following the regulations in Italy (D.M. 116192), Europe (O.J. of E.C. L 358/1 12/18/1986).

### 4.2. Controlled Cortical Impact (CCI) Experimental Traumatic Brain Injury TBI

Mice were anesthetized under intraperitoneal (i.p.) ketamine (2.6 mg/kg body weight) and xylazine (0.16 mg/kg body weight). TBI was made in mice by a controlled cortical impactor (CCI) as previously described [3]. In brief, a craniotomy was made in the right hemisphere, between the sagittal suture and the coronal ridge, with a micro-motor handpiece and drill. The resulting bone flap was removed, and a cortical contusion was produced on the exposed cortex using the controlled impactor device (Impact OneTM Stereotaxic, Leica, Milan, Italy). The impact tip was positioned and lowered over the craniotomy place until it touched the dura mater. Then, the piston was retracted, and the impact tip was advanced farther to produce a brain injury of moderate severity for mice (piston diameter: 4 mm; cortical contusion depth: 3 mm; impact velocity: 1.5 m/s). After TBI was induced, the skin incision was closed with nylon sutures, and 2% lidocaine jelly was applied to the lesion place to reduce any possible discomfort.

### 4.3. Experimental Groups

Mice were casually distributed into the following groups: (n = 10/each group)

**TBI:** mice were subjected to CCI, and vehicle (saline, 300 mL i.p.) was administered at 1 h after injury induction.

**TBI+bevacizumab:** mice were subjected to CCI, and a single i.p. of bevacizumab (Avastin, Genentech/Roche, 75 μg/300 μL) was administered at 1 h after injury induction.

**Sham+vehicle:** mice were exposed to the same surgical procedures as the above group (anesthesia and craniotomy), except the impact tip was not applied, and vehicle (saline, 300 mL i.p.) was administered at 1 h after craniotomy.

**Sham+bevacizumab:** mice were subjected to the same surgical procedures as the above group (anesthesia and craniotomy), except the impact tip was not applied, and bevacizumab (Avastin, Genentech/Roche, 75 μg/300 μL) was administered at 1 h after craniotomy (data are not shown in results as no significant differences were observed with the Sham + vehicle).

The bevacizumab dose (75 μg/300 μL) was chosen according to the dose used in other studies present in the literature [46].

In the first set of experiments, mice were sacrificed 24 h after TBI and brains were collected and processed for histology and biochemical analyzes.

In the second set of experiments, mice were sacrificed 6 weeks after TBI and hearts, including aortic valves, were collected and processed for histology and biochemical analyzes.

### 4.4. Behavioral Testing

All animals underwent behavioral examinations 24 h after CCI to assess motor and cognitive deficits. Three different reputable expert observers, blind to the animal’s state of injury, conducted the behavioral tests. All the behavioral studies were performed in a blinded fashion. The tests are described below:

#### 4.4.1. Rotarod Test (RT)

For the RT, the mice underwent a week of training before induction of TBI. The rotarod treadmill (Accuscan, Inc., Columbus, OH, USA) made it possible to assess motor balance and coordination. The test was performed as previously described by Campolo et al. [47]. The data were estimated by averaging the scores (total time spent on the treadmill divided by five trials) for each animal during training and testing days. In detail, the maximum score assigned to an animal was set at 60 s. During training, the animals were subjected to five tests per day and the criterion was declared achieved when the animals obtained a score of 60 s in three consecutive tests. For the tests, the animals were subjected to three tests and the average score of these three tests was used as the individual rotarod score.

#### 4.4.2. Elevated Body Swing Test (EBST)

No training was required for EBST. This test provided a motor asymmetry parameter and involved manipulating the mice by the tail and recording the direction of the distorted body oscillations. The EBST consisted of 20 trials with the number of ipsilateral and contralateral oscillations to the injured hemisphere recorded and expressed in % to determine the distorted oscillation activity [48].

### 4.5. Measurement of Edema (Brain Water Content)

At 24 h post-CCI, the animals were euthanized to determine edema via brain water content. The cortices, excluding the cerebellum, were rapidly removed, and the contralateral and ipsilateral hemispheres were weighed separately. Each hemisphere was dried at 60 °C for 72 h, and the dry weight was determined. The water content was calculated in the ipsilateral hemisphere as % Water content = (wet weight − dry weight)/wet weight × 100.

### 4.6. Histological Analysis

For the first set of experiments, coronal sections of the brain (7 μm) were fixed, cut, stained with Hematoxylin and Eosin (Bio-Optica, Milan, Italy) and analyzed by a qualified histopathologist using an optical microscope associated with an imaging system (Leica Microsystems SpA, Milan, Italy). The damaged neurons were counted and the histopathologic changes of the gray matter were scored on a six-point scale [49,50]: 0 = no lesion; 1 = gray matter contained 1 to 5 eosinophilic neurons; 2 = gray matter contained 5 to 10 eosinophilic neurons; 3 = gray matter contained more than 10 eosinophilic neurons; 4 = small infarction (less than one-third of the gray matter area); 5 = moderate infarction (one third to one half of the gray matter area); 6 = large infarction (more than half of the gray matter area).

For the second set of experiments, hearts embedded in paraffin were cut to 7 μm, obtaining sections at the level of the aortic sinus. These sections were subsequently stained with hematoxylin and eosin for the quantification of the lesion area normalized by the adjacent medial area of the aorta to be checked for possible tangential section [51]. The lesion region was defined as the area between the endothelial cell layer and the internal elastic lamina. The scores from all the sections of each brain and heart were averaged to give a final score for an individual mouse. All the histological studies were performed in a blinded fashion. The degree of fibrosis was evaluated by Masson’s trichrome method according to the manufacturer’s protocol (Bio-Optica, Milan, Italy) [52]. The quantity of fibrosis was evaluated as the percentage of the fibrotic area (blue staining) and quantified using image analysis software (Image J 1.8.0). Two impartial observers reported the severity of histological damage following standardized criteria.

### 4.7. Western Blot Analysis

Cytosolic and nuclear proteins were extracted from the ipsilateral hemisphere of brain and heart tissues as previously described [53,54]. The following primary antibodies were used in PBS with 5% *w*/*v* non-fat dried milk and 0.1% Tween-20 at 4 °C O/N: anti-VEGF (SCB, 1:500, #sc57496, D.B.A, Milan, Italy); anti-ZO-1 (SCB, 1:500; #sc-33725; D.B.A, Milan, Italy); anti-Occludin (SCB, 1:500; #sc-133255; D.B.A, Milan, Italy); anti-Claudin-5 (SCB, 1:500; #sc-374221; D.B.A, Milan, Italy); anti-ICAM1 (SCB, 1:500; #sc-8439; D.B.A, Milan, Italy); anti-IKB-α (SCB, 1:500, #sc-1643, D.B.A, Milan, Italy); anti-NF-kB (SCB; 1:500 #sc8008, D.B.A, Milan, Italy); anti-GFAP (SCB, 1:500; #sc-33673; D.B.A, Milan, Italy); anti-Iba-1 (SCB, 1:500; #sc-32725; D.B.A, Milan, Italy); anti-Bax (SCB, 1:500; #sc-7480; D.B.A, Milan, Italy); anti-Bcl-2 (SCB, 1:500; #sc-7382; D.B.A, Milan, Italy). Membranes were incubated with peroxidase-conjugated bovine anti-mouse IgG secondary antibody or peroxidase-conjugated goat anti-rabbit IgG (Jackson ImmunoResearch, West Grove, PA, USA; 1:2000) for 1 h at room temperature. Anti-β-actin or anti-lamin A/C (D.B.A, Milan, Italy) antibodies were used as controls. Protein expression was analyzed as previously reported [55].

### 4.8. Cytokine and Soluble E-Selectin Measurements

In the ipsilateral hemisphere of the brain tissues obtained after sacrifice, TNF-α (Ray Bio ELISA Kit Mouse TNF-alpha, Norcross, GA, USA), IL-1β and CCL2 (R&D Systems, Milan, Italy) levels were evaluated using a colorimetric commercial kit [56,57].

Plasma samples were collected 6 weeks after TBI. Circulating concentrations of soluble E-selectin were measured with an ELISA kit following the manufacturers’ instructions (R&D Systems, Minneapolis, MN, USA) [27,58].

### 4.9. Statistical Analysis

All values in the figures and text are expressed as the mean ± standard deviation (SD) of N observations. For the in vivo studies, N represents the number of animals studied. In experiments involving histology, the figures shown are representative of at least three experiments performed on different days on tissue sections collected from all animals in each group. The results were analyzed by one-way ANOVA followed by a Bonferroni post hoc test for multiple comparisons. A *p*-value of less than 0.05 was considered significant.

## Data Availability

The datasets used in the current study are available from the corresponding author on reasonable request.
